# Entosis Acts as a Novel Way within Sertoli Cells to Eliminate Spermatozoa in Seminiferous Tubule

**DOI:** 10.3389/fphys.2017.00361

**Published:** 2017-05-30

**Authors:** Nisar Ahmed, Ping Yang, Yufei Huang, Hong Chen, Tengfei Liu, Lingling Wang, Fazul Nabi, Yi Liu, Qiusheng Chen

**Affiliations:** ^1^Laboratory of Animal Cell Biology and Embryology, College of Veterinary Medicine, Nanjing Agricultural UniversityNanjing, China; ^2^Faculty of Veterinary and Animal Sciences, Lasbela University of Agriculture, Water and Marine Sciences (LUAWMS)Uthal, Pakistan

**Keywords:** *in vivo* entosis, spermatozoa, Sertoli cell, hibernation, Chinese soft-shelled turtle

## Abstract

The present study was designed to investigate the hypothesis that *in vivo* entosis is a novel pathway for eliminating spermatozoa in the seminiferous tubules (ST) during hibernation of the Chinese soft-shelled turtle. Western blot analysis revealed that the expression of LAMP1 in the testis was significantly higher during hibernation than that during non-hibernation. Immunohistochemistry reaction showed that LAMP1-positive substance was distributed within the Sertoli cells of the testis. Further examination by transmission electron microscopy (TEM), many degraded spermatozoa being enwrapped within large entotic vacuoles in Sertoli cells. The nucleus and the flagellum of the spermatozoa were shown to be decomposed and digested inside entotic vacuoles within Sertoli cells. More than two spermatozoa heads were always observed in each internalized vacuoles. Deserving note is that, a number of different autophagosomes, including initial autophagic vesicles and degradative autophagic vesicles were found inside the entotic vacuoles of the Sertoli cells during hibernation. At the end of hibernation, entotic vacuoles and their autophagosomes disappeared, and numerous large lipid droplets (LDs) appeared within the Sertoli cells. Adherens junctions were apparent between Sertoli cells and developing germ cells, which is the ultrastructural basis of entosis. Taken together, the results presented here show that in the turtle: (1) entosis with internal autophagosomes can take place within normal body cells during hibernation; (2) spermatozoa, as a highly differentiated cell can be internalized and degraded within Sertoli cell by entosis *in vivo*, which is in favor of the next reproductive cycle in the turtle.

## Introduction

Chinese soft-shelled turtles (*Pelodiscus sinensis*) are one of the common species of reptiles, and are widely distributed in China. This species is famous for its economic and pharmacologic value, and hence, they are subject to harvesting pressure (Xiangkun et al., 2008). This turtle has a typical pattern of hibernation with a special process of germ cell development. In our previous study, spermatogenesis in the soft-shelled turtle was shown to start in May and continue through summer and early autumn, with spermiation being complete by early November. The majority of male germ cells progressed through the phases of spermatogenesis as a single cohort within the seminiferous tubules (ST) (Zhang et al., 2007, 2015; Nainan et al., 2010). After spermiation in late autumn, residual spermatozoa on the ST must be eliminated during hibernation (the non-spermatogenic phase), which is an essential program for the next reproductive season.

Entosis is a novel mechanism of non-apoptotic death of the internalized cells; consequently, it eliminates the dead cells outside of their normal microenvironment. Although observations of entosis have been reported from last several years, but the underlying cellular and molecular mechanism is still largely unknown (Li et al., 2015a). Through the process of entosis cells become internalized into neighboring cells, forming what are called “cell-in-cell” structures. This mechanism is frequently observed within the exudates that contain metastatic carcinoma cells, or *in vitro* culture cells. In the past, it is largely unknown that the entosis also occurs among normal cells within the body (Florey et al., 2010). However, a recent study reports showed that the blastocyst trophoblasts engulf uterine epithelial cells by entosis (Li et al., 2015a). So combining the spatial relationships of spermatozoa and Sertoli cells, the present study brings forward a hypothesis that *in vivo* entosis is a novel pathway for eliminating spermatozoa in the ST during hibernation of the Chinese soft-shelled turtle.

Different from entosis, autophagy is another pathway of cell clearance happened in the interior of the cell. Through autophagy, intracellular substrates are engulfed into double-membrane vesicles called autophagosomes, which deliver material to lysosomes for digestion (Florey and Overholtzer, 2012). Autophagosomes can enwrap material non-specifically, during bulk turnover of cytoplasm, enabling the survival of nutrient-deprived cells, or specifically, to target damaged organelles, protein aggregates, or specific proteins for lysosomal degradation or secretion (Yang and Klionsky, 2010). Recently, it is reported that proteins from the autophagy pathway control lysosome fusion to entotic vacuoles in an autophagy-independent manner (Florey and Overholtzer, 2012), suggesting that there may be a relationship between autophagy (degrading intracellular material) and entosis (degrading extracellular material). To elucidate the cellular mechanism of the elimination of male germ cells in ST during hibernation, the present study investigated cytological evidence of spermatozoa clearance by entosis within Sertoli cells using western blot analysis, immunohistochemistry, and transmission electron microscopy (TEM).

## Materials and methods

### Experimental animals and ethical approval

A total of 50 adult male soft-shelled turtles, *Pelodiscus sinensis*, were captured from the Baoying aqua farm in Jiangsu province of China; ten turtles were examined each month (February, May, July, October, and December) in 2014. The hibernation season for this species is always from November to the following April, while the non-hibernation (reproductive) season is from May to October in Jiangsu province of China. Animals were anesthetized with an intraperitoneal administration of sodium pentobarbital (20 mg/kg) and killed by cervical dislocation. Testes samples were collected and quickly prepared for subsequent tests. One testis from each animal was prepared for Western blot tests, and the other was prepared for immunohistochemistry tests and transmission electron microscopy examination. The sample procedures were conducted in accordance with the guidelines of the Animal Research Institute Committee of Nanjing Agriculture University. All the protocols were approved by the Science and Technology Agency of Jiangsu Province. The approval ID is SYXK (SU) 2011-0036. All precautions were taken to minimize animal suffering.

### Transmission electron microscopy

Testes samples were cut into 1 mm^3^ blocks and immersed in 2.5% glutaraldehyde in 0.01M phosphate- buffered saline (PBS) (4°C, pH 7.4,) for 24 h. The samples were post-fixed in 1% osmium tetroxide solution for 1 h at 37°C, after rinsing in PBS. Samples were dehydrated in ascending concentrations of ethyl alcohol, infiltrated with a propylene oxide-Araldite mixture and embedded in Araldite. The ultrathin sections of tissue (50 nm) were mounted on copper grids, and contrasted with uranyl acetate and lead citrate for 20 min each step. Finally, sections were examined and photographed with a transmission electron microscope H-7650.

### Immunohistochemistry of lamp-1

Generally, samples were fixed in the formal saline solution and then embedded in paraffin. Serially sectioned were done (6 μm) and mounted on poly-L-lysine-treated glass slides. After deparaffinization, sections were treated with 3% H_2_O_2_ at 37°C for 10 min to block the activity of endogenous peroxidase. After three washes in PBS, the slides were incubated in a citrate buffer solution in boiling water for 3 min. After washing three times with PBS, the slides were incubated in the 5% BSA solution from the SABC immunohistochemistry Kit (Boster, China) for 30 min in a humidified chamber to block nonspecific binding. Sections were incubated separately at 4°C for 15 h with either 200 μg/mL of LAMP-1 antibody (NB120-19294, Novus Biologicals, Oakville, CA). After washing, the sections were incubated with a goat anti-rabbit IgG antibody (Boster, China) for 1 h. The sections were incubated with SABC for 30 min. The peroxidase activity was revealed on the sections using 0.02% (w/v) 3–30 diaminobenzidinetetrahydrochloride (DAB) containing 0.001% (v/v) H_2_O_2_ in 0.05 M Tris-HCl (pH 7.6). The sections were washed with water and finally, counterstained with hematoxylin. As a control group, the first antibody was replaced with PBS.

### Western blot analysis of lamp-1

Five testis samples from different turtles in each month (February, May, July, October, and December) in 2014 were homogenized in ice-cold RIPA's buffer (25 mMTris/HCl (pH 7.6), 150 mMNaCl, 1% sodium deoxycholate, 1% Nonidet-P40, 0.1% SDS, 0.05 mM PMSF). The protein concentration was quantified by BCA protein assay (Thermo Fisher Scientific, Rockford, USA). Equal amount of each proteins samples (40 μg/lane) was subjected to electrophoresis on 10% SDS-PAGE and transferred to polyvinylidene di-fluoride (Millipore, Bedford, MA) membranes. After blocking in 5% fat-free dry milk, membranes were incubated with an LAMP-1 antibody (21997-1-AP, Proteintech Group, Chicago, IL) at a dilution of 1:1,000 for 12 h at 4°C. After washing, the membrane was incubated with peroxidase-linked goat anti-rabbit IgG (1:5,000, BS13278, Bioworld Technology Inc., Louis Park, MN) for 2 h. Bound antibodies were detected using the ECL detection system (Vazyme Biotech, China). Immunoreactive bands were quantified with Quantity One software (Bio-Rad Laboratories).

### Statistical analysis

Data from western blot test are expressed as the mean ± SEM. Data were analyzed using SPSS software version 14.0 with one way ANOVA followed by Duncan's test. The differences were considered significant at the level of *P* < 0.05.

## Results

### Lysosomal membrane protein (LAMP1) was expressed in the testis, being specifically located inside sertoli cells

**Figure 1:**
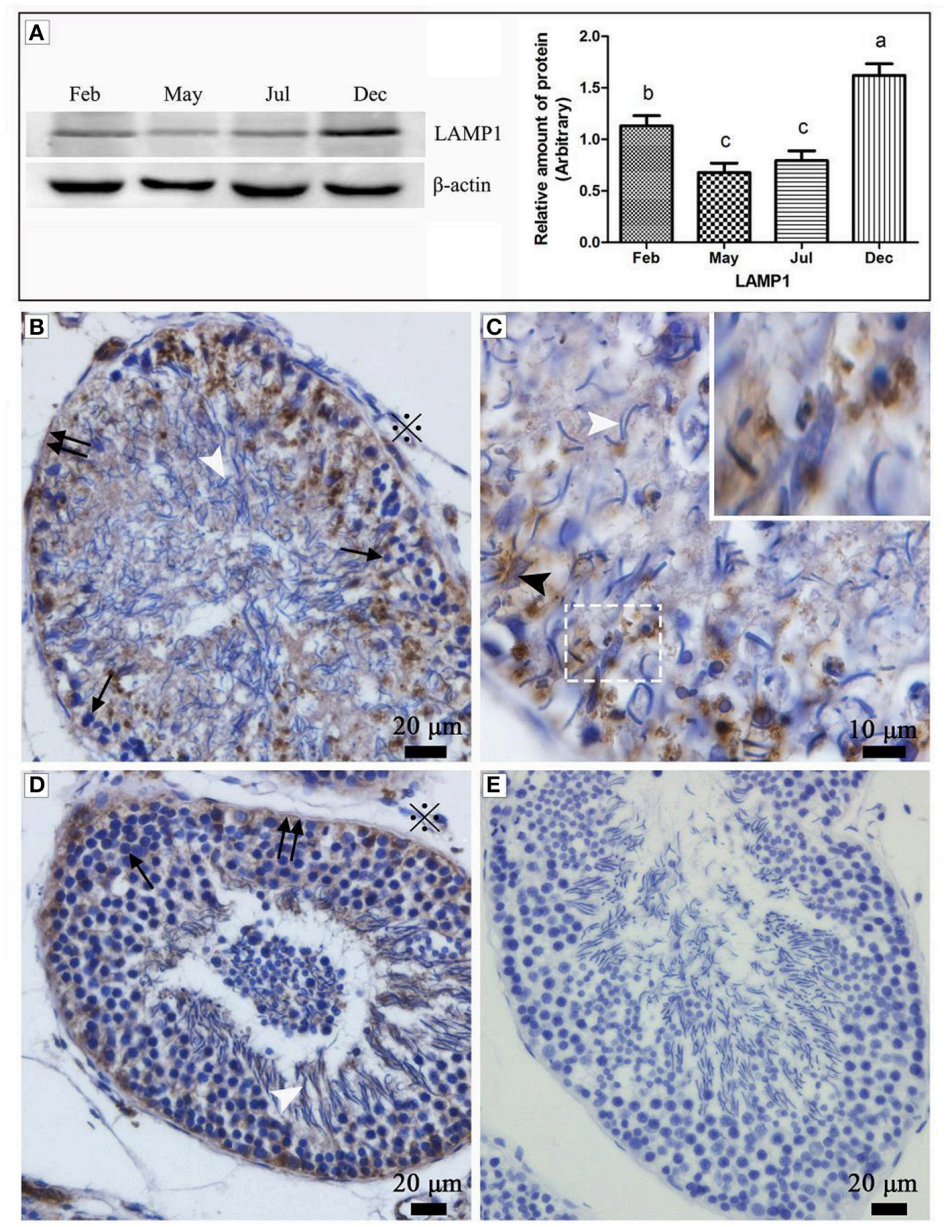
Western blot analysis and immunohistochemistry reaction of the LAMP1 protein in the testis of *P. sinensis*. The histogram represents a densitometric analysis of the immunoblots in **(A)** by western blot analysis of LAMP1 protein. Each bar shows the mean ± SEM of triplicate assays. Averages with the same superscripted letters are not significantly different at *P* ≤ 0.05. The positive substance was brown in color by immunohistochemistry reaction: **(B,C)** Feb; **(D)** July; **(E)** negative control. Germ cells (black arrow), spermatozoon (white arrow head), nucleus of Sertoli cell (black arrow head), interstitial tissue of testis (snowflake), basement membrane of ST (double black arrow). Scale bar = 20 μm **(A,C,D)** and 10 μm **(B)**.

Western blot results showed that the expression of LAMP1 within the turtle testis was highly significant during hibernation (samples in Dec. and Feb.) than the non-hibernation period (samples in May and Jul.) (*P* < 0.05) (Figure 1A). LAMP1 is a well-established as a lysosomal marker. Immunohistochemistry further detected that LAMP-1 was observed in Sertoli cells and surround some spermatozoa head, and that the localization was stronger in February and December (hibernation) than in May and July (non-hibernation) (Figures 1B–E).

### Various entotic vacuoles occurred within sertoli cells during hibernation

**Figure 2:**
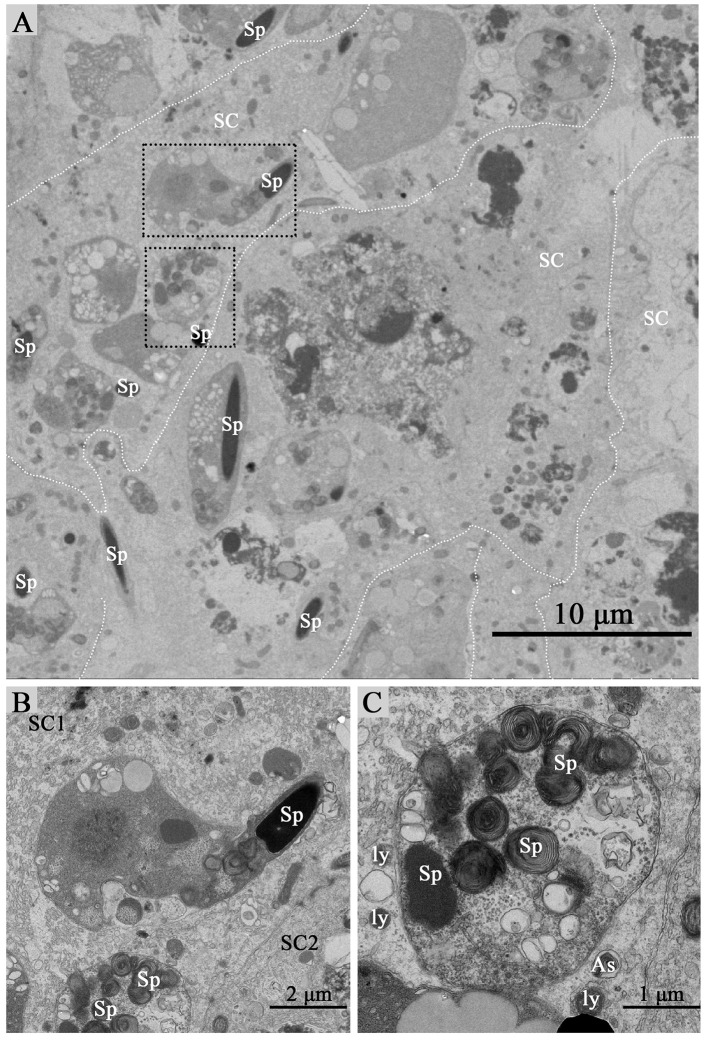
TEM photograph of Sertoli cells of the ST. Some integrity spermatozoa internalized within Sertoli cell. The dotted line shows the boundaries between the Sertoli cells **(A)**. **(B,C)** Show higher magnification of the boxed area in **(A)**. A living spermatozoon with remaining adherens junction internalized of Sertoli cell **(B)**, and the Sertoli cell degraded the internalized cell through autophagy/lysosomal **(C)**. Spermatozoa (Sp), Sertoli cell (SC), lysosome (ly), and autophagosome (As). Scale bar = 10 μm **(A)** 2 μm **(B)**, and 1 μm **(C)**.

**Figure 3:**
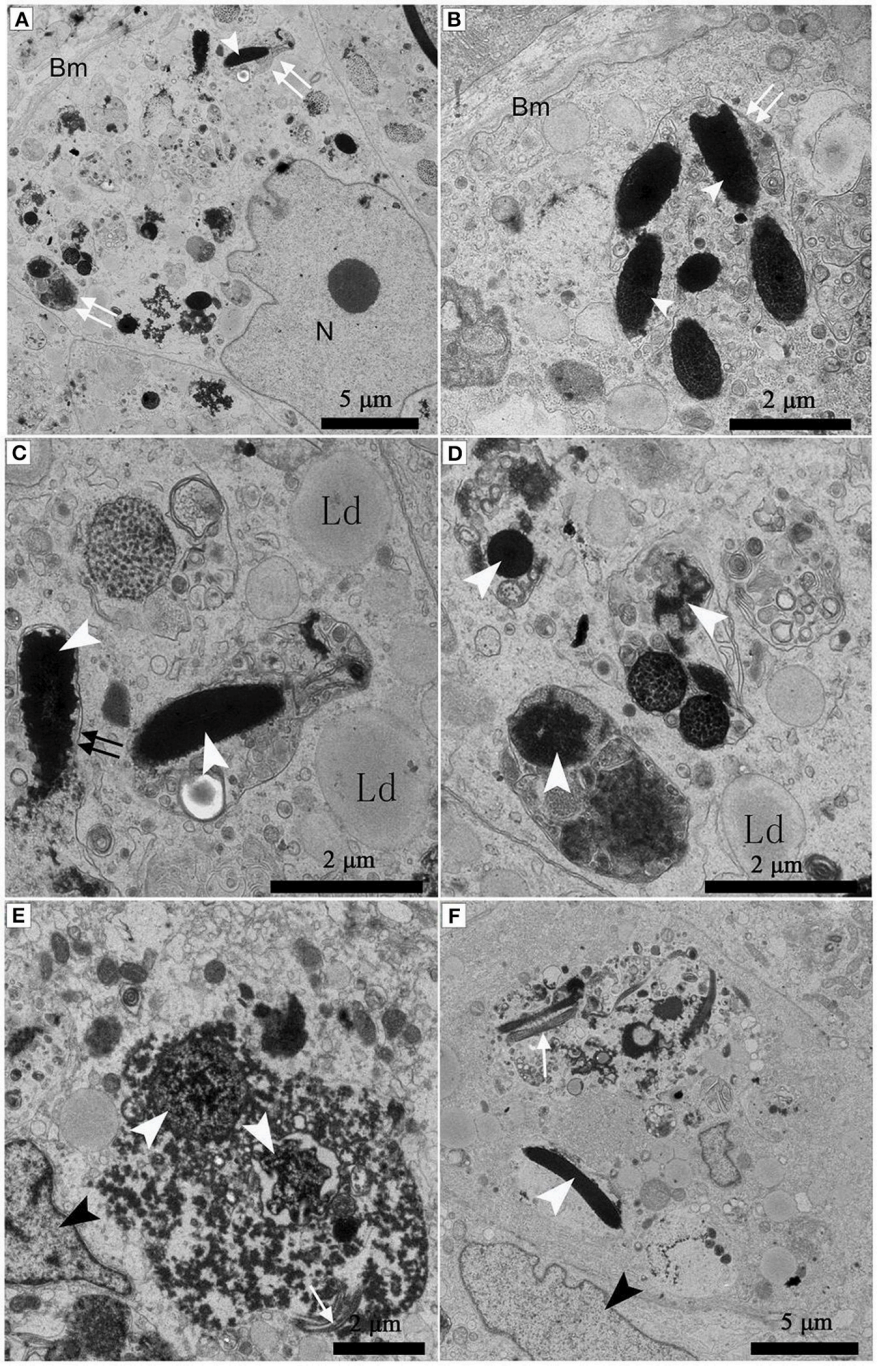
TEM photograph of Sertoli cells of the ST. Many entotic vacuoles of different stages were frequently observed within Sertoli cells in Feb; **(A)**. Several spermatozoa were wrapped within each entotic vacuole in Dec **(B)**. Different internalized entotic vacuoles within Sertoli cells were seen in Feb **(C–E)** and in Dec **(F)**. Entotic vacuole (double white arrow), nucleus of the Sertoli cell (N), basal membrane (Bm), several sperm heads (white arrowhead) within one entotic vacuole; Nucleus of the spermatozoon (white arrowhead), flagellum of the spermatozoon (black arrow), nucleus of the Sertoli cell (black arrowhead), lipid droplet (Ld). Scale bar = 5 μm **(A,F)** and 2 μm **(B–E)**.

Under TEM, some living spermatozoa with normal morphology were seen within the Sertoli cell (Figure 2). And many entotic vacuoles of different stages were frequently observed within Sertoli cells (Figure 3A) in samples taken during hibernation months, and some lysosome and autophagosome surround these entotic vacuoles, which corresponded with the results of LAMP-1 immune staining and western blot tests. Spermatozoa heads in different orientations were always inside internalized entotic vacuoles. Several spermatozoa were always wrapped within each entotic vacuole (Figures 2C, 3B). During entosis of a spermatozoa within a Sertoli cell, the internalized spermatozoa nucleus became loose and granulated (Figures 3C–E), while the flagellum of the spermatozoa was gradually disassembled during hibernation (Figures 3E,F).

**Figure 4:**
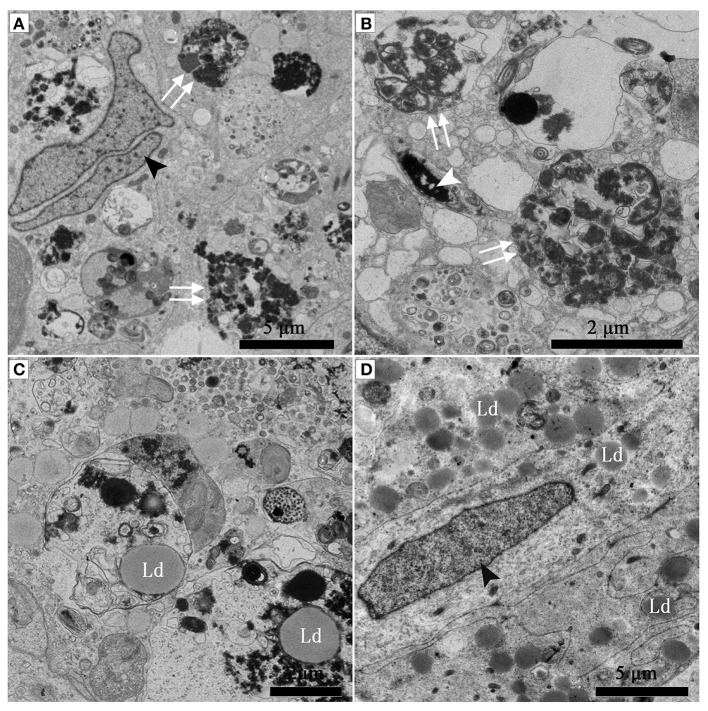
TEM photograph of Sertoli cell of the ST. (A–C) in Feb; **(D)** in May. Nucleus of the spermatozoon (white arrowhead), degraded entotic vacuole (double white arrow), lipid droplet (Ld), nucleus of the Sertoli cell (black arrowhead). Scale bar = 5 μm **(A,D)** and 2 μm **(B,C)**.

Many entotic vacuoles were degraded and digested (Figures 4A–C) in February during hibernation, while numerous large lipid droplets (Figure 4D), rather than entotic vacuoles, arose within Sertoli cells at the beginning of non-hibernation in May.

**Figure 5:**
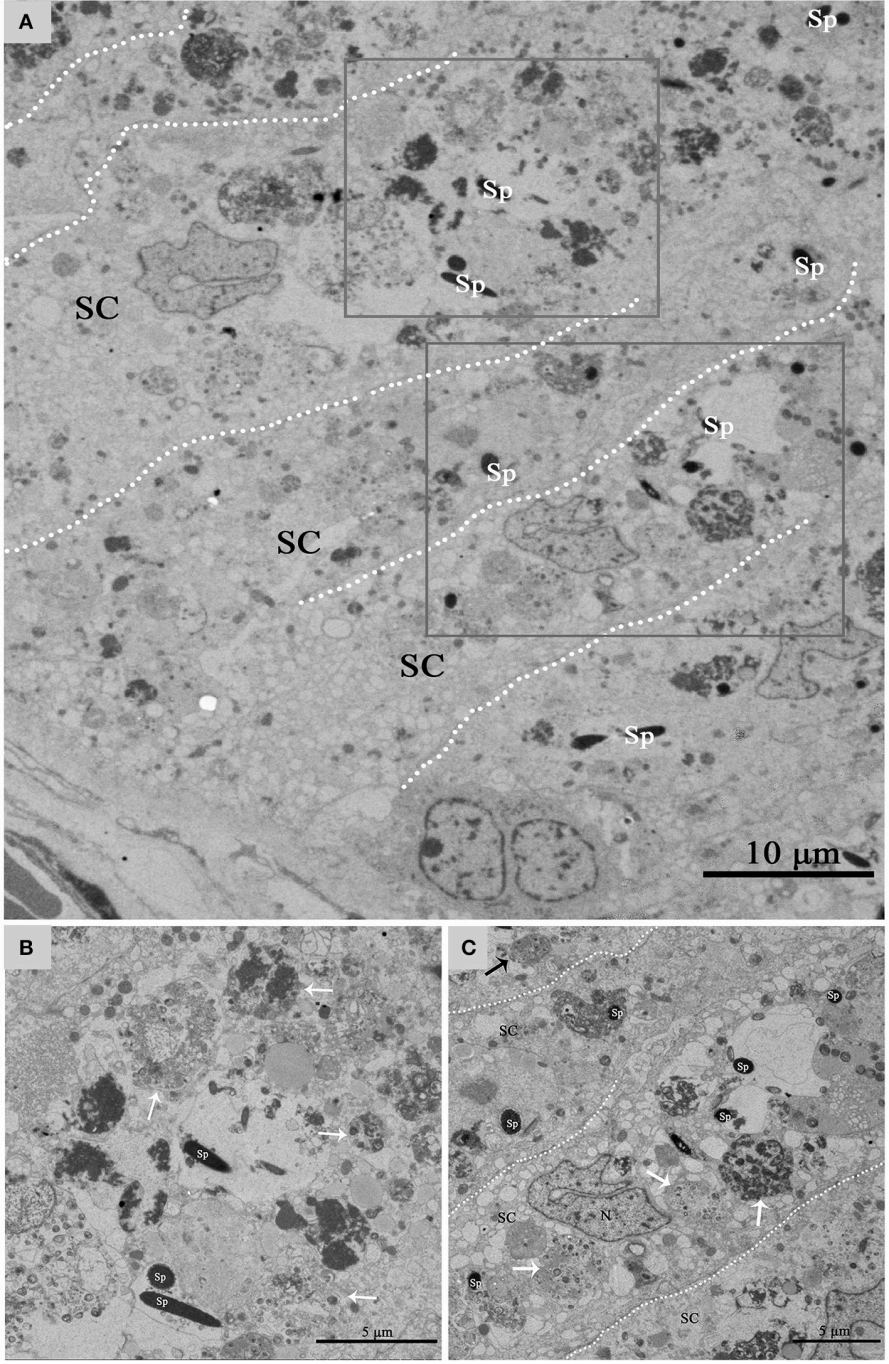
TEM photograph of Sertoli cell of the ST. **(A)** Shows a large number of living cells (spermatozoa) within the luminal surface of Sertoli cell in hibernation. **(B,C)** Show higher magnification of the boxed area in **(A)**. The process of degradation and digestion of entotic vacuoles from the luminal surface **(A)** to middle of Sertoli cell **(B)**. The dotted line shows the boundaries between the Sertoli cells. Spermatozoa (Sp), Sertoli cell (SC). Scale bar = 10 μm **(A)** and 5 μm **(B,C)**.

Figure 5 shows a direct evidence that we can seen some living cells, within the luminal surface of Sertoli cell, and can observed the entotic vacuoles of different stages from the luminal surface to the basal surface of Sertoli cell in hibernation months. The entotic vacuoles and their degradation decreases within Sertoli cell from the luminal surface to the basal membrane of seminiferous tubules. In contrast, it was hardly to see the entotic vacuoles and living spermatozoa inside Sertoli cells in non-hibernation months (Supplementary Figure 1).

### Numerous different autophagosomes were observed by TEM within the entotic vacuoles of sertoli cells

**Figure 6:**
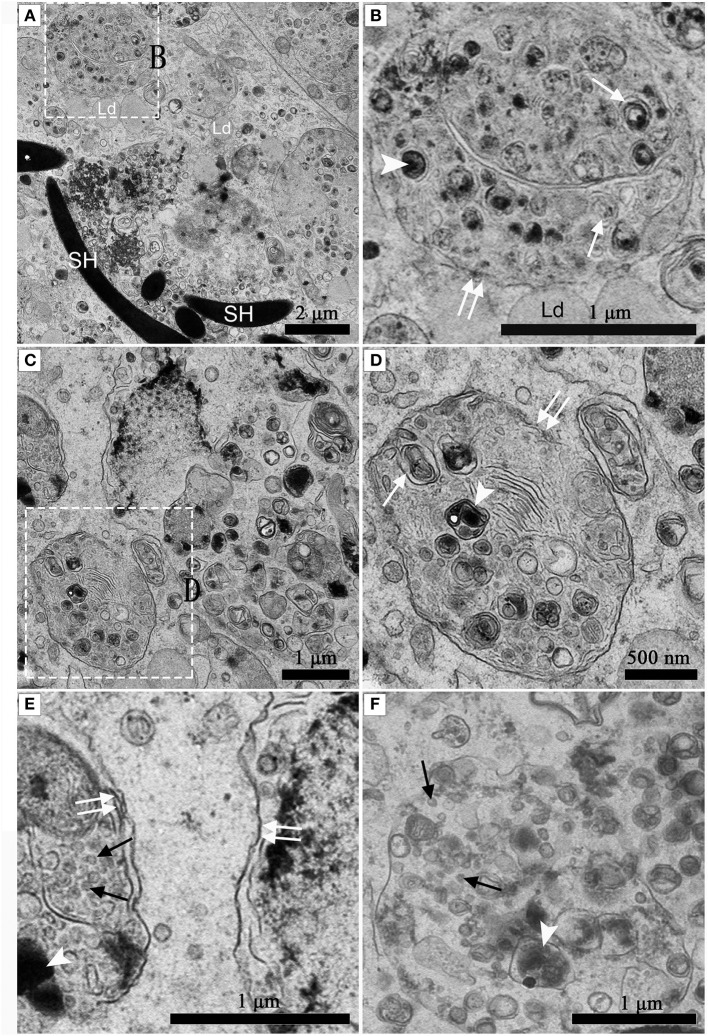
TEM photograph of Sertoli cell in the ST. **(A,B)** in Feb; **(C,D)** in Dec; **(E,F)** in Feb. **(B)** shows higher magnification of the boxed area in **(A)**. **(D)** Shows higher magnification of the boxed area in **(C)**. Entotic vacuole (double white arrow), initial autophagic vesicles (AVis) (white arrow), degradative autophagic vesicles (AVds) (white arrowhead), Lipid droplet (Ld), sperm head (SH), small endosome vesicles (black arrow). Scale bar = 2 μm **(A)**, 1 μm **(B,C,E,F)**, and 500 nm **(D)**.

Under TEM, numerous early/initial autophagic vesicles (AVis) and late/degradative autophagic vesicles (AVds) were observed within the entotic vacuoles of Sertoli cells (Figure 6). AVis with double-membranes contained morphologically intact cytoplasmic material (Figures 6B,D), and AVds contained partially degraded cytoplasmic material (Figures 6D,F). The entotic vacuoles also fused with multivesicular endosomes, as indicated by the contents of numerous small vesicles (Figures 6E,F).

### Clear adherens junctions were observed between developing germ cells and sertoli cells during spermiogenesis in july and in october before hibernation. numerous primary lysosomes were distributed within the sertoli cells during non-hibernation season

**Figure 7:**
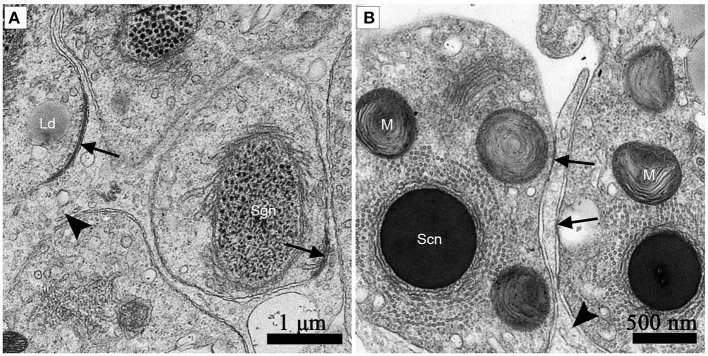
TEM photograph of the ST. Adherens junctions were apparent between spermatids with granular nuclei and Sertoli cells in July, during spermiogenesis **(A)**. Before spermiation, the number and intensity of adherens junctions decreased between spermatozoa with compact nuclei and the processes of Sertoli cells in October, before hibernation **(B)**. Adherens junctions (black arrow), lipid droplets (Ld), processes of Sertoli cells (black arrowhead), spermatozoon with granular nucleus (Sg), spermatozoa with a compact nucleus (Sc), mitochondrion (M). Scale bar = 1 μm **(A)** and 500 nm **(B)**.

Adherens junctions were apparent between spermatids with granular nuclei and Sertoli cells in July, during spermiogenesis (Figure 7A). Before spermiation, the number and intensity of adherens junctions decreased between spermatozoa with compact nuclei and the processes of Sertoli cells in October, before hibernation (Figure 7B).

**Figure 8:**
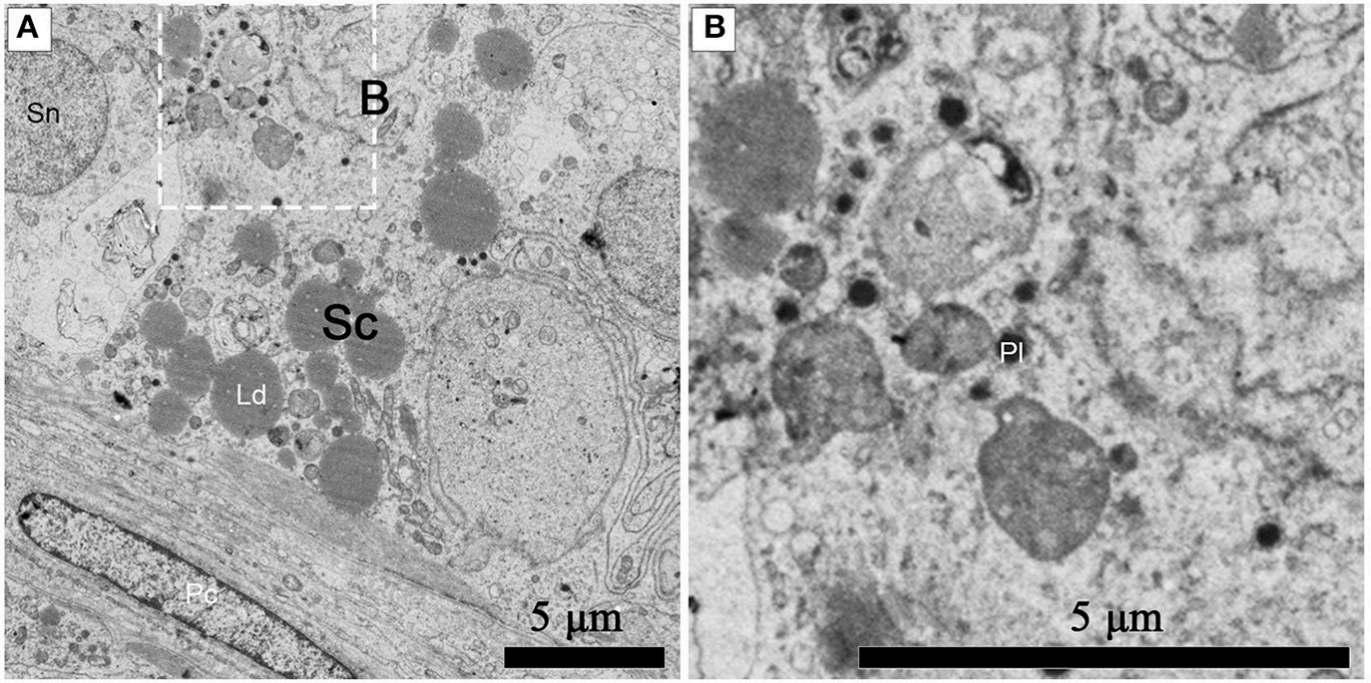
TEM photograph of a Sertoli cell in the ST. Numerous primary lysosomes were observed within Sertoli cells during spermiogenesis in July **(A)**, **(B)** shows higher magnification of the boxed area. Sertoli cell (Sc), primary lysosome (Pl), lipid droplets (Ld), spermatogonium nucleus (Sn), peritubular contractile cell (Pc). Scale bar = 5 μm **(A,B)**.

Numerous primary lysosomes were observed within Sertoli cells during spermiogenesis in July (Figures 8A,B).

## Discussion

Entosis is a novel process, whereby cells become internalized into neighboring cells, hence, forming “cell-in-cell” structures. The main outcome of these internalized cells is non-apoptotic cell death through lysosome-mediated degradation. Mostly, homotypic cell-in-cell structures are observed among sibling tumor cells, while heterotypic phenomena occurs between tumor cells and immune cells, previously termed “emperipolesis” (Wang et al., 2013). As the main outcome of entosis is the death of the internalized cell, it may represent a novel mechanism for eliminating cells outside of their normal microenvironment (Florey et al., 2010). *In vivo*, entosis disrupts normal cytokinesis, resulting in aneuploidy in human breast cancer cells (Krajcovic et al., 2011). Entosis may also have a role in normal physiological processes (Li et al., 2015b). So there is an expecting thing about heterotypic cell-in-cell structures related to *in vivo* bulk cell elimination under physiological conditions. A recent study reported that blastocyst trophoblasts engulf uterine epithelial cells by entosis, but lacking of the process of degradation and digestion of entotic vacuoles (Li et al., 2015a). Morphology observated on the microstructure and ultrstructure has always been credited as the most effective means in cells identification and classifications, based on the evaluation of the integral structures and local details of target cells. Here, we present obvious evidence that spermatozoa invade Sertoli cells during the hibernation season, when the degraded germ cells need to be cleared for the next spermatogenesis cycle within the ST of the this turtle. Furthermore, our results show that the spermatozoa head and flagellum can be degraded and digested within the entotic vacuoles in Sertoli cells, although spermatozoa are terminally differentiated with compact heads and a motive flagellums. Previous studies from our laboratory on spermatogenesis have been performed in Chinese soft-shelled turtle (*Pelodiscus sinensis*), which mainly focused on the apoptosis and proliferation of germ cells (Zhang et al., 2008, 2015). As we know, the contents in the nucleus of spermatozoa are so highly dense that apoptosis related genes are difficult to be expressed. Apoptotic bodies were therefore came from spermatids, not spermatozoa. Phagocytosis is always related to the dead cells; however a large number of living cells (spermatozoa) were present in the Sertoli cell. Meanwhile, in our manuscript we even pointed out that the existence of autophagosome in entotic vacuole, which can't be found in dead cells. Moreover, our western blot and immunohistochemistry findings showed a high expression of LAMP1during hibernation period than the non-hibernation period. However, LAMP1 is a specific marker for lysosme, but recently it has been shown that LAMP1 is transiently recruited to the late entotic vacuole (Florey et al., 2011). These interesting phenomena have driven us to find the fate of these residual spermatozoa during hibernation. So the present study mainly focused on the role of entosis in the elimination of spermatozoa in testis.

Autophagy is an intercellular self-degradation process through double-membrane organelles known as autophagosome, whereby cellular organelles or proteins are phagocytosed due to metabolic stress (Levine and Klionsky, 2004). Recently, autophagy has also been linked to the normal death process (Montero et al., 2010), and it is an evolutionarily conserved physiological process (Lin et al., 2012; Zhang et al., 2012). In critical situations the process of phagocytosis of dying cells is overwhelmed by enormous cell death occurred by autophagy through the lysosomal degradation (Debnath et al., 2005). However, in mammalian culture cells autophagic cell death is observed (Aburto et al., 2012) and it accompanies with other degradation processes during development, where massive cell death occurred (Montero et al., 2010), cell death by autophagy is infrequent in vertebrates (Shen et al., 2012). It is speculate that the autophagy serves as an important mechanism of cell clearance within the body. In the ST of the Chinese soft shelled turtle, large number of degrading spermatozoa must be removed during the hibernation season, and the reproductive epithelium must be remodeled for the following reproductive cycle. In the present study, under TEM, numerous different autophagosomes within entotic vacuoles were observed inside Sertoli cells of the turtle ST, indicating that autophagy can take place when male germ cells are eliminated by entosis during the hibernation season in this species. Recently evidence has emerged that proteins from the autophagy pathway control lysosome fusion to macroendocytic vacuoles, in an autophagy-independent manner, suggesting that these proteins control well-known pathway of intracellular substrate turnover also control the more general degradation of extracellular substrates in a variety of cell systems *in vitro* (Martinez et al., 2011; Takahashi et al., 2011; Florey and Overholtzer, 2012). To the best of our knowledge, the present study shows for the first cytological evidence about the many different autophagosomes develop inside the entotic vacuoles *in vivo*. This suggests that autophagy plays a large role in entosis when massive germ cells need to be degraded and eliminated within Sertoli cells of the turtle during the hibernation season. No evidence is available regarding cross-talk between autophagy and entosis, and further investigation is needed to clarify the molecular mechanism of how entosis and autophagy act together to eliminate large numbers of cells (Zhang et al., 2012).

The vacuoles formed by entotic cell engulfment undergo sequential steps of maturation leading to fusion with lysosomes. Lysosomal hydrolytic enzymes result in the death of living cells engulfed during entosis (Florey et al., 2011). Although the overall sequence of maturation events that controls lysosomal fusion is well described (Kinchen and Ravichandran, 2008; Flannagan et al., 2012), the subsequent stages that follow the degradation of engulfed cargo after fusion are poorly understood. The process of entosis entails sequential steps of maturation, leading to the fusion of lysosomes, which digest internalized cargo. After digestion, nutrients must be exported to the cytosol, and vacuole membranes must be processed by mechanisms that remain poorly defined (Krajcovic et al., 2013). On the other hand, autophagic pathway components associate with LDs, and autophagy regulates hepatic lipid stores *in vivo* (Singh et al., 2009). In Chinese soft-shelled turtle, numerous primary lysosomes were distributed in Sertoli cells during spermiogenesis in July, and their fusion with the entotic vacuole and autophagosome took place during the hibernation season. The present study provided sufficient evidence of the process of eliminating spermatozoa within Sertoli cells, thus, we hypothesized that there may be a relationship between entosis (degrading spermatozoa depending on the role of Sertoli cells) and autophagy (degrading intracellular material of ntotic vacuoles). At the end of hibernation there arose numerous LDs, instead of entotic vacuoles or autophagosomes. This suggests that formation of LDs may be one of the subsequent stages after engulfed cargo is degraded by entosis and autophagy, and that LDs may serve as a source of nutrition and energy for the next spermatogenesis cycle in this species.

Entosis leads to the physical elimination of the “loser” cells, which usually succumb to death of cells through non-apoptotic way (Kroemer and Perfettini, 2014). During entosis, internalized cells are always enwrapped by entotic vacuoles within the target cells (Wang et al., 2013). Commonly, this occurs between various tumor cells such as breast, carcinoma cells, cervical, or colon (Overholtzer et al., 2007), suggesting that loss of integrin-mediated adhesion may promote cell-in-cell invasion. A matrix-detached target cell invading a neighboring host cell requires the formation of adherens junctions (Yuan and Kroemer, 2010). Apparent adherens junctions were present between spermatids, the precursor cells of spermatozoa and Sertoli cells in the ST during spermiogenesis (Figure 7A). These junctions were less frequent before spermiation (Figure 7B). This implies that a trigger structure exists for entosis between germ cells and their supporting cells, Sertoli cells. Therefore, the degraded spermatozoa in the ST might move into the Sertoli cells after spermiation, when they detach from the ST epithelium during the hibernation season. This *in vivo* cytological evidence supports the viewpoint that the “loss of adherens junctions may promote cell-in-cell invasion (entosis)” (Purvanov et al., 2014).

## Author contributions

The authors have made the following declarations about their contributions: Conceived and designed the experiments: QC, NA, and PY. Performed the experiments: NA, PY, YH, TL, LW, FN. Analyzed the data: NA, PY, HC, and YL. Contributed reagents/materials/analysis tools: NA, PY, YH, TL. Wrote the paper: NA and PY.

### Conflict of interest statement

The authors declare that the research was conducted in the absence of any commercial or financial relationships that could be construed as a potential conflict of interest.

## Supplementary material

Supplementary Figure 1 **TEM photograph of the ST during non hibernation period**. Sertoli cell contains no any entotic vacuoles or spermatozoa. The dotted line shows the boundaries between the Sertoli cells. Nucleus (N). Scale bar = 10 μm.
