# Supplementary figures and images for: Entosis Acts as a Novel Way within Sertoli Cells to Eliminate Spermatozoa in Seminiferous Tubule
Source: Front Physiol. 2017 May 30;8:361. doi: 10.3389/fphys.2017.00361 (PMC5447735; doi:10.3389/fphys.2017.00361)

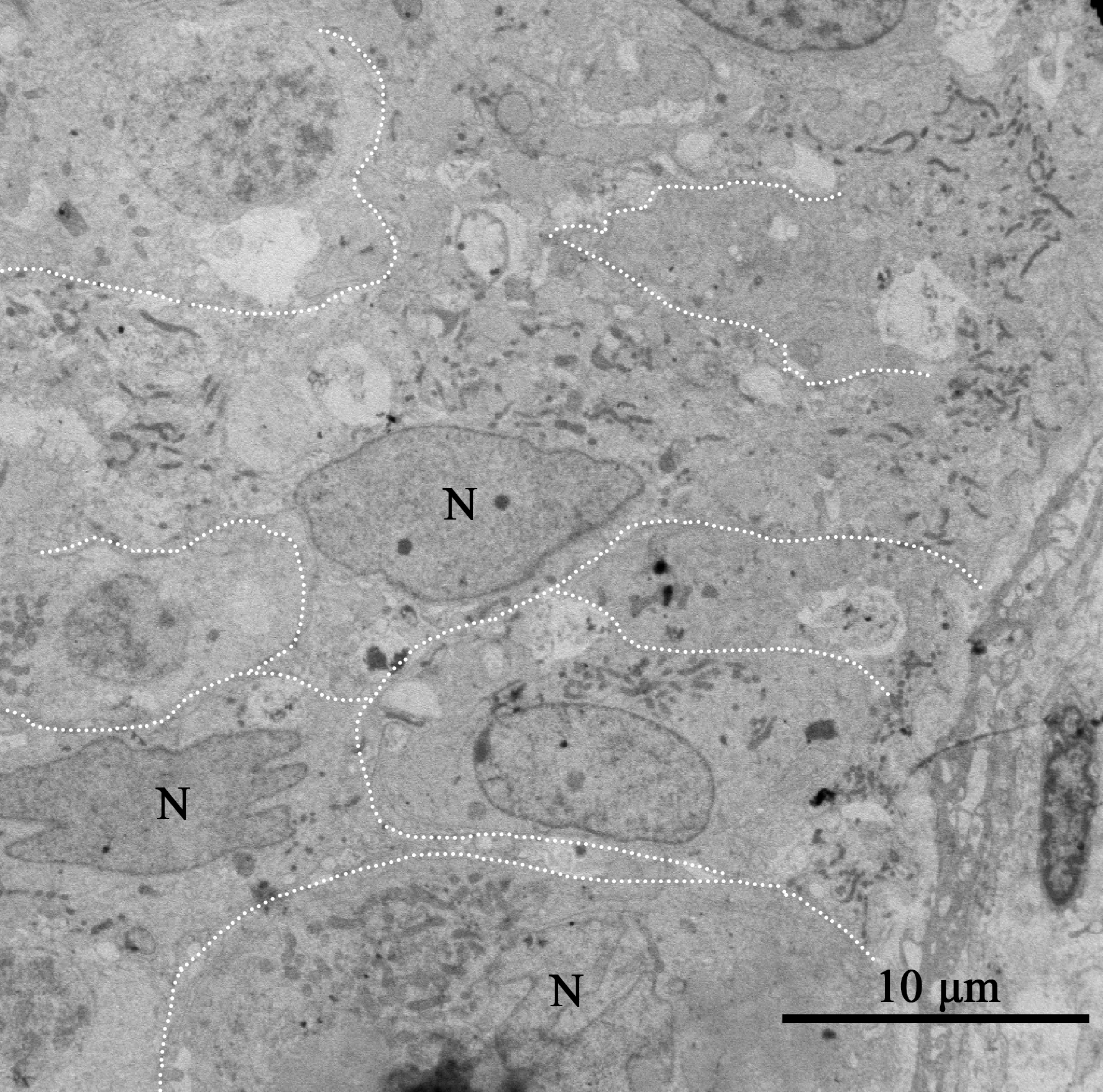

Supplement: Supplementary Figure 1 — TEM photograph of the ST during non hibernation period. Sertoli cell contains no any entotic vacuoles or spermatozoa. The dotted line shows the boundaries between the Sertoli cells. Nucleus (N). Scale bar = 10 μm. [file Image1.JPEG]
